# Supplementary material for: Polymorphisms in Pfkelch13 domains before and after the introduction of artemisinin-based combination therapy in Southwest Nigeria
Source: PLoS One. 2025 Mar 31;20(3):e0316479. doi: 10.1371/journal.pone.0316479 (PMC11957316; doi:10.1371/journal.pone.0316479)

Samples: 12663
Bases: 1070
Average spacing: 12

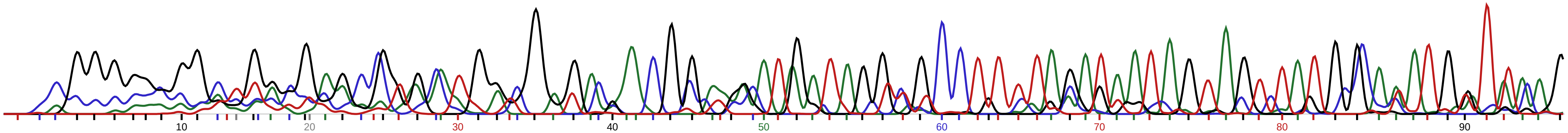

T C C G G G G G G G G C T - G C A C G - A G C T G T G C A T G G T C G A G A C G A A C G G A T G C A T G A T A G G T G C C T G T T T A G A T A A T A G T A G T T A T G G A A A T G G T T A A G

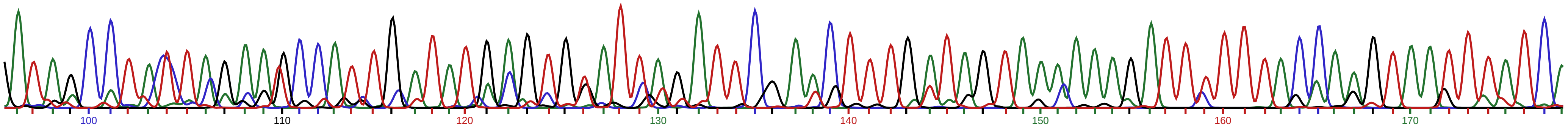

A T A G C C T A T T A G A A G C C A T T G A T A T G A G T G T A T T A G A T T C G A A C T T T G A T A G T A A A A A A G A T T T T T T A C C A A G T A A T T T A T C A

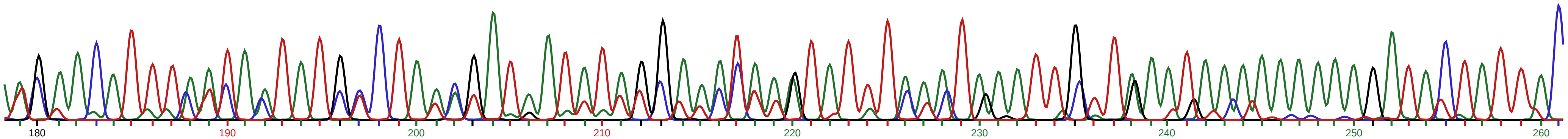

A G A A C A T T T A A T A A T A T G C C T A A A G A T A A T A T A G G A A A T A A A T A T T T A A A T A A A T T G T T A A A T A A A A A A A A A G A T A C T A T T A C

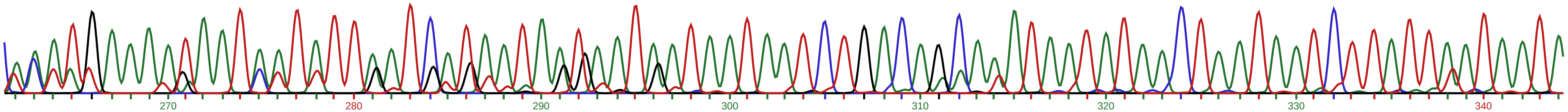

A A A T G A A A A T A A T A A T A T T A A T C A T A A T A A T A A T A A T A A T A A T A A T C T G A C A G C A A A T A A T A T A A C T A A T A A T C T T A T T A A T A A T A

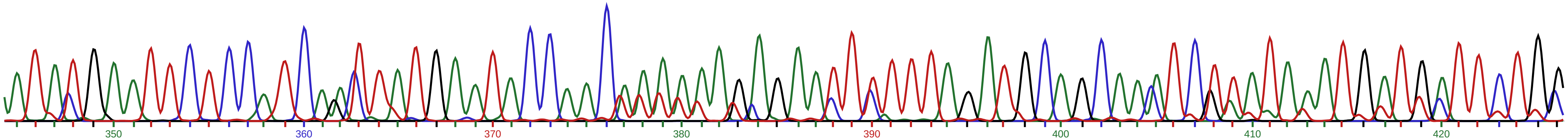

A T A T G A A T T C T C C A T C A A T T A T G A A T A C C A A C A A A A A A G A G A A T T T T T T A G A T G C A G C A A A T C T T A T A A A T G A T G A T T C T G G

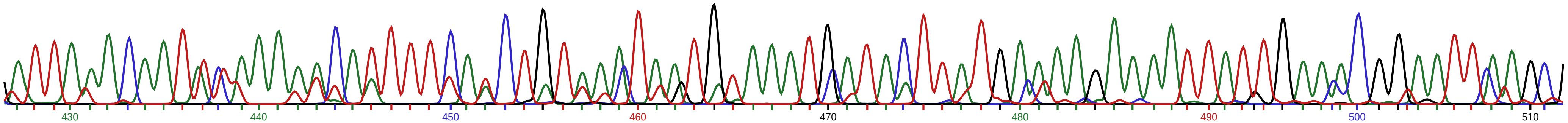

A T T A A A C A A T T C A A A A A C A T T T T C A A C T G T A A A T A A T G T A A A T G A T A C T T A T G A A A A G A A A A T T A T G T G A A C A C G G T A A T T A A G C

Samples: 12663
Bases: 1070
Average spacing: 12

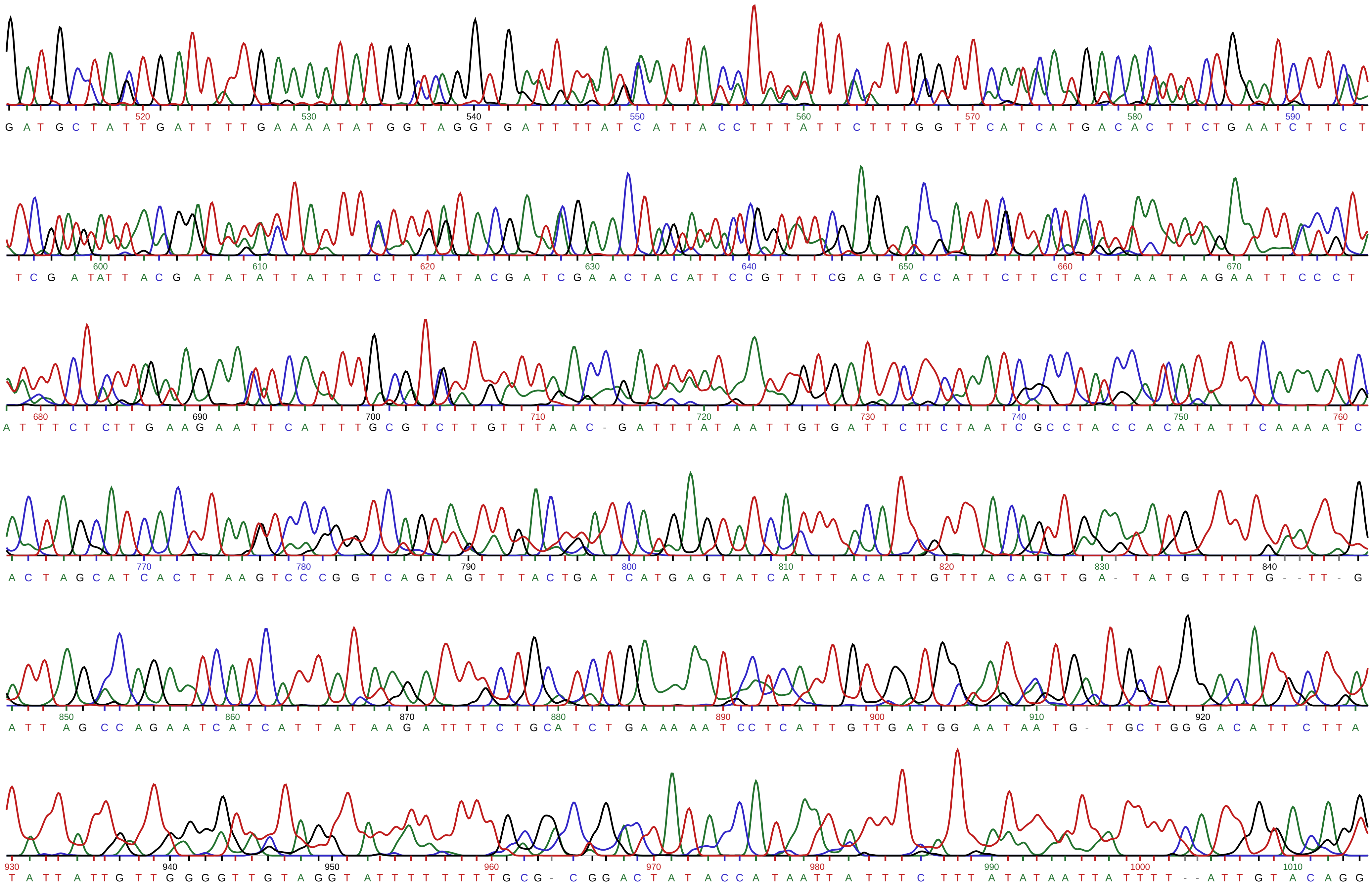

Samples: 12663
Bases: 1070
Average spacing: 12

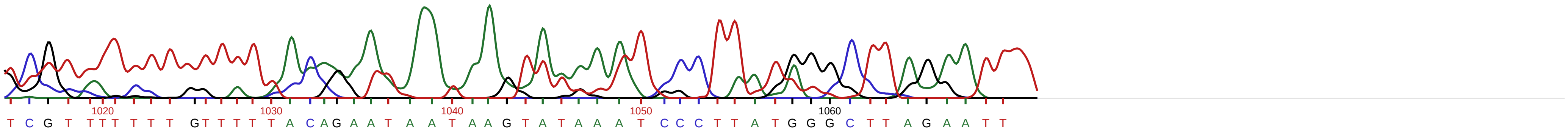

Supplement: Supporting information 2 — (ZIP) [file pone.0316479.s002.zip › 054KN2F_PREMIX_Plate_KELCH2_A09.pdf]
